# Erbium laser‐assisted ceramic debonding: a scoping review

**DOI:** 10.1111/jopr.13613

**Published:** 2022-11-10

**Authors:** Janina Golob Deeb, Kinga Grzech‐Leśniak, Erica R. Brody, Jacek Matys, Sompop Bencharit

**Affiliations:** ^1^ Department of Periodontics School of Dentistry Virginia Commonwealth University Richmond Virginia; ^2^ Laser Laboratory Department of Oral Surgery Wroclaw Medical University Wroclaw Poland; ^3^ Health Sciences Library Virginia Commonwealth University Richmond Virginia; ^4^ Department of Oral and Craniofacial Molecular Biology Philips Institute for Oral Health Research School of Dentistry Virginia Commonwealth University Richmond Virginia; ^5^ Department of Biomedical Engineering College of Engineering Virginia Commonwealth University Richmond Virginia

**Keywords:** Ceramic crown, debonding, Erbium lasers, Er,Cr:YSGG laser, Er:YAG laser

## Abstract

**Purpose:**

Removal of ceramic restorations and appliances can be time consuming, invasive, and inconvenient. Erbium lasers offer an alternative noninvasive method for debonding of ceramic appliances. This paper aims to provide a comprehensive review of current literature on the effectiveness of erbium lasers for removal of ceramic restorations and appliances from natural teeth and dental implants.

**Methods:**

A comprehensive search of 7 databases, including Medline (Ovid), Embase, Dentistry and Oral Sciences Source (DOSS), Web of Science, Cochrane Library, and ProQuest Dissertations and Theses was performed. The inclusion and exclusion criteria were agreed prior to the literature search. Two reviewers independently screened the title and abstract. A third reviewer then broke the tie, if any. The selected articles then underwent full text review and the data was extracted.

**Results:**

The search identified 4117 unique articles published through June 10, 2021. Studies were assessed and categorized based on the type of restoration/appliance, type of abutment, type of laser, laser settings, efficacy of debonding, and pulpal temperature rise. Thirty‐eight full‐text articles were reviewed for inclusion. Time for ceramic debonding varies depending on the type of restorations and materials. Removal of zirconia crowns from teeth and implant abutments requires a longer period of time compared to lithium disilicate crowns. Temperature increases were reported as 5.5 degrees or less. Laser setting and laser type affect the debonding time and the increase in temperature. Examinations of debonded ceramics demonstrated no known structural damages resulting from laser applications.

**Conclusions:**

Erbium lasers are effective noninvasive tools to remove all ceramic restorations/appliances from natural teeth and implant abutments without causing harm to abutments. Laser‐assisted debonding should be considered as a viable alternative to rotary instrumentation for ceramic crowns; however, clinical studies of erbium‐assisted ceramic retrieval are needed.

All‐ceramic restorations and orthodontic brackets have been popularized for their esthetic advantages, biocompatibility, and predictable durability.^1^, ^2^ The longevity of ceramic restorations on natural abutment teeth is influenced by external damage or fracture of restoration, caries risk, marginal integrity, microleakage of the luting cement, or endodontic therapy after the placement of restorations. This warrants the removal of the ceramic restoration from the tooth.^3^, ^4^ Ceramic implant restorations may have to be removed for esthetic or functional reasons, including abutment screw loosening or breaking. The conventional removal procedure of ceramic restorations is often performed by sectioning with rotary instruments using diamond or tungsten carbide burs. This traditional removal method is inconvenient and damages the integrity of the restoration.^5^ The removal procedure can be lengthy and result in damage to the underlying natural tooth or implant abutment.^6^, ^7^, ^8^ Removing all‐ceramic restorations or orthodontic brackets from natural teeth can also be challenging due to the similarity between the color of the cement and underlying tooth structure.^9^, ^10^

The use of erbium lasers has been explored as an alternative method for debonding of ceramic appliances from natural teeth and implants,^2^, ^11^, ^12^, ^13^ including removal of translucent restorative materials such as composite restorations,^14^, ^15^ fiber reinforced composite posts,^16^ veneers,^11^, ^12^, ^13^ and orthodontic brackets.^10^, ^17^, ^18^, ^19^ The light emitted by erbium lasers, such as Erbium, Chromium‐doped Yttrium Scandium Gallium Garnet (Er,Cr:YSGG) and Erbium‐doped Yttrium Aluminum Garnet (Er:YAG) lasers carrying wavelengths of 2780 nm and 2940 nm, respectively, can be transmitted through the translucent ceramic materials and selectively absorbed by water molecules and residual monomers in luting cements, thus resulting in the vaporization of these molecules and debonding of the cement.^5^, ^16^, ^20^ This method offers many advantages, however its efficiency can be affected by several clinical operating factors including chemical composition, shade and thickness of the cement, type, shade, opacity, and thickness of the ceramic restoration, and laser parameters such as power, pulse duration, and frequency.^6^, ^10^, ^16^, ^21^ Recent studies have demonstrated predictability in debonding of ceramic restorations with both erbium lasers.^20^ Several concerns with the laser‐assisted crown debonding remain around heat generation and thermal injury to the adjacent tissues. An increase in pulpal temperature by 5.5°C can cause irreversible damage to the pulp tissue.^22^ An increased osseous temperature by 10°C can cause bone damage.^23^ Temperature increases to 6°C can damage periodontal ligament.^24^

The objectives of this study are therefore (1) to provide a summary of the current comprehensive literature on erbium family laser‐assisted ceramic restoration and orthodontic appliance debonding from teeth and implant abutments; (2) to provide insight into the erbium laser settings, clinical outcomes, and possible complications; and (3) identify knowledge gaps, scope a body of literature, and clarify current concepts of Erbium lasers in ceramic debonding

## METHODS

### Identification of relevant literature

A systematic search was conducted in the following databases on July 31, 2020 and updated on June 10, 2021: Medline (Ovid), Embase, Dentistry and Oral Sciences Source (DOSS), Web of Science, Cochrane Library, and ProQuest Dissertations and Theses. Keywords and controlled vocabulary were used to search for the concepts of dental debonding and erbium‐doped yttrium, aluminum, and garnet (Er:YAG) and erbium, chromium‐doped yttrium, scandium, gallium and garnet (Er,Cr:YSGG) lasers. No date restrictions were used, however, results were limited to those published in English. The detailed search strategy used in Medline (Ovid) is provided in Table 1. The PRISMA checklist is provided as Supplementary Table 1.

**TABLE 1:** Detailed search strategies

Medline/OVID	(Dental debonding/ OR (exp dental implants/ or exp dental prosthesis/ or exp ceramics/ or exp dental cements/ or exp orthodontics/ OR ((dental AND implant*) or crown or crowns or inlay or inlays or onlay or onlays or veneer or veneers or restoration* or abutment* or bracket* or dental porcelain or zirconium or ceramics or ceramic or dental cement* or braces or bridge or laminate or laminates or posts or lithium disilicate or enamel or dentin or dentistry or orthodont*).mp.) AND (lasers, solid state/ OR (solid‐state laser* or Er:YAG laser* or “laser” or “Er: YAG laser*” or erbium laser* or ER laser* or Er‐YAG laser* or Er YAG laser* or “erbium‐doped” or “erbium‐doped yttrium aluminium garnet” or erbium YAG laser* or “ER,CR:YSGG” or “ER, CR:YSGG” or “ER, CR: YSGG” or “ER,CR: YSGG” or “erbium, chromium: yttrium‐scandium‐gallium‐garnet” or biolase).mp.)) NOT (hair removal OR eye or retina* or cornea*)
Embase	(Dental Debonding/ OR ((exp tooth implant/ or exp tooth prosthesis/ or exp ceramics/ or exp tooth cement/ or orthodontics/ OR ((dental and implant*) or crown or crowns or inlay or inlays or onlay or onlays or veneer or veneers or restoration* or abutment* or bracket* or dental porcelain or zirconium or ceramics or ceramic or dental cement* or braces or bridge or laminate or laminates or posts or lithium disilicate or enamel or dentin or dentistry or orthodont*).mp.) AND (remov* or debond* or ablation or ablate* or retriev*).mp.) AND (exp solid state laser/ OR (solid‐state laser* or Er:YAG laser* or “laser” or “Er: YAG laser*” or erbium laser* or ER laser* or Er‐YAG laser* or Er YAG laser* or “erbium‐doped” or “erbium‐doped yttrium aluminium garnet” or erbium YAG laser* or “ER,CR:YSGG” or “ER, CR:YSGG” or “ER, CR: YSGG” or “ER,CR: YSGG” or “erbium, chromium: yttrium‐scandium‐gallium‐garnet” or biolase).mp.) NOT (hair removal OR eye or retina* or cornea*)
MEDLINE Database	(“Dental Debonding”[Mesh] OR (“dental implants”[mesh] or “dental prosthesis”[mesh] or “ceramics”[mesh] or “dental cements”[mesh] or “orthodontics”[mesh] OR “dental implant*”[tw] or “crown”[tw] or “crowns”[tw] or “inlay”[tw] or “inlays”[tw] or “onlay”[tw] or “onlays”[tw] or “veneer” [tw] or “veneers” [tw] or “restoration*” [tw] or “abutment*”[tw] or “bracket*”[tw] or “dental porcelain”[tw] or “zirconium”[tw] or “ceramics”[tw] or “ceramic”[tw] or “dental cement*”[tw] or “braces”[tw] or “bridge”[tw] or “laminate”[tw] or “laminates”[tw] or “posts”[tw] or “lithium disilicate”[tw] or “enamel”[tw] or “dentin”[tw] or “dentistry”[tw] or “orthodont*”[tw]) AND (“remov*”[tw] or “debond*”[tw] or “ablation”[tw] or “ablate*”[tw] or “retriev*”[tw])) AND (“lasers, solid‐state”[mesh] OR “solid‐state laser*”[tw] or “Er:YAG laser*”[tw] or “laser”[tw] or “Er: YAG laser*”[tw] or “erbium laser*”[tw] or “ER laser*”[tw] or “Er‐YAG laser*”[tw] or “Er YAG laser*”[tw] or “erbium‐doped”[tw] or “erbium‐doped yttrium aluminium garnet”[tw] or “erbium YAG laser*”[tw] or “ER,CR:YSGG”[tw] or “ER, CR:YSGG”[tw] or “ER, CR: YSGG”[tw] or “ER,CR: YSGG”[tw] or “erbium, chromium: yttrium‐scandium‐gallium‐garnet”[tw] or “biolase”[tw]) NOT (“hair removal”[tw] OR “eye”[tw] or “retina*”[tw] or “cornea*”[tw])
Web of Science	(“dental debonding” OR ((orthodontic* OR (crown or crowns or inlay or inlays or onlay or onlays or veneer or veneers or restoration* or abutment* or bracket* or dental porcelain or zirconium or laminate or laminates or lithium disilicate or enamel or dentin or implant* or prosthes?s OR ceramic* or cement*) AND (dental OR dentistry)) AND (remov* or debond* or ablation or ablate* or retriev*)) AND (“solid‐state laser*” OR (“Er:YAG laser*” or “Er: YAG laser*” or “erbium laser*” or “ER laser*” or “Er‐YAG laser*” or “Er YAG laser*” or “erbium‐doped” or “erbium‐doped yttrium aluminium garnet” or “erbium YAG laser*” or “ER,CR:YSGG” or “ER, CR:YSGG” or “ER, CR: YSGG” or “ER,CR: YSGG” or “erbium, chromium: yttrium‐scandium‐gallium‐garnet” or biolase))
ProQuest Dissertation and Theses	noft(“dental debonding” OR (((orthodontic* OR crown or crowns or inlay or inlays or onlay or onlays or veneer or veneers or restoration* or abutment* or bracket* or dental porcelain or zirconium or laminate or laminates or lithium disilicate or enamel or dentin or implant* or prosthesis OR prostheses OR ceramic* or cement*) AND (dental OR dentistry)) AND (remov* OR debond* OR ablation OR ablate OR retriev*))) AND noft(“solid‐state laser*” OR “Er:YAG laser*” or “Er: YAG laser*” or “erbium laser*” or “ER laser*” or “Er‐YAG laser*” or “Er YAG laser*” or “erbium‐doped” or “erbium‐doped yttrium aluminium garnet” or “erbium YAG laser*” or “ER,CR:YSGG” or “ER, CR:YSGG” or “ER, CR: YSGG” or “ER,CR: YSGG” or “erbium, chromium: yttrium‐scandium‐gallium‐garnet” or biolase)
DOSS	(DE “Debonding” OR ((DE “DENTAL implants”) or DE “PROSTHODONTICS” or (DE “DENTAL ceramics”) or DE “DENTAL cements” OR (DE “ORTHODONTICS”) OR (crown or crowns or inlay or inlays or onlay or onlays or veneer or veneers or restoration* or abutment* or bracket* or dental porcelain or zirconium or ceramics or ceramic or dental cement* or braces or bridge or laminate or laminates or posts or lithium disilicate or enamel or dentin or dentistry or orthodont*) AND (remov* or debond* or ablation or ablate* or retriev*)) AND ((DE “LASERS in dentistry”)) OR (DE “SOLID‐state lasers”) OR (solid‐state laser* or Er:YAG laser* or “laser” or “Er: YAG laser*” or erbium laser* or ER laser* or Er‐YAG laser* or Er YAG laser* or “erbium‐doped” or “erbium‐doped yttrium aluminium garnet” or erbium YAG laser* or “ER,CR:YSGG” or “ER, CR:YSGG” or “ER, CR: YSGG” or “ER,CR: YSGG” or “erbium, chromium: yttrium‐scandium‐gallium‐garnet” or biolase))
Cochrane Library	(“Dental Debonding”[Mesh] OR (“dental implants”[mesh] or “dental prosthesis”[mesh] or “ceramics”[mesh] or “dental cements”[mesh] or “orthodontics”[mesh] OR “dental implant*”[tw] or “crown”[tw] or “crowns”[tw] or “inlay”[tw] or “inlays”[tw] or “onlay”[tw] or “onlays”[tw] or “veneer” [tw] or “veneers” [tw] or “restoration*” [tw] or “abutment*”[tw] or “bracket*”[tw] or “dental porcelain”[tw] or “zirconium”[tw] or “ceramics”[tw] or “ceramic”[tw] or “dental cement*”[tw] or “braces”[tw] or “bridge”[tw] or “laminate”[tw] or “laminates”[tw] or “posts”[tw] or “lithium disilicate”[tw] or “enamel”[tw] or “dentin”[tw] or “dentistry”[tw] or “orthodont*”[tw]) AND (“remov*”[tw] or “debond*”[tw] or “ablation”[tw] or “ablate*”[tw] or “retriev*”[tw])) AND (“lasers, solid‐state”[mesh] OR “solid‐state laser*”[tw] or “Er:YAG laser*”[tw] or “laser”[tw] or “Er: YAG laser*”[tw] or “erbium laser*”[tw] or “ER laser*”[tw] or “Er‐YAG laser*”[tw] or “Er YAG laser*”[tw] or “erbium‐doped”[tw] or “erbium‐doped yttrium aluminium garnet”[tw] or “erbium YAG laser*”[tw] or “ER,CR:YSGG”[tw] or “ER, CR:YSGG”[tw] or “ER, CR: YSGG”[tw] or “ER,CR: YSGG”[tw] or “erbium, chromium: yttrium‐scandium‐gallium‐garnet”[tw] or “biolase”[tw]) NOT (“hair removal”[tw] OR “eye”[tw] or “retina*”[tw] or “cornea*”[tw])

Limits: English Language.

### Study selection and data collection

Study selection was conducted in two phases. First, 3 abstract reviewers (J.G.D., J.M., K.G.L.) independently reviewed titles and abstracts of citations obtained from the systematic literature search. Articles marked for inclusion by at least 2 reviewers were retained for the second phase of full‐text review. Three reviewers examined abstracts for inclusion. Two reviewers examined all full‐text articles independently (J.M., K.G.L.), and a third reviewer (J.G.D.) was consulted to resolve any disagreements.

Inclusion criteria included in vitro and in vivo studies of Er:YAG/Er,Cr:YSGG laser debonding (removal, retrieval) of veneers, laminates, lithium‐disilicate, zirconium‐oxide crowns, prosthetic crowns, fixed bridges, implant crowns, brackets. Exclusion criteria included non‐English papers, review, clinical reports, opinions, editorial papers; nonerbium laser or no laser; only adhesive removal; only resin composite debonding, or debonding of fiber posts, glass fibers; and laser surface preparation before bonding or debonding procedures.

Two authors (J.M. and K.G.L) reviewed the final included full text articles and collected the following information: sample size, study group composition, sample type (e.g. type of abutment, type of restoration/appliance etc.), laser parameters, radiation exposure/retrieval time, and temperature changes. A third author (J.G.D.) reviewed and compiled the data from full text articles into summary. The fourth author (S.B.) verified the validity of the charted data and finalized the results.

## Results

### Study selection and characteristics of the included studies

**FIGURE 1:**
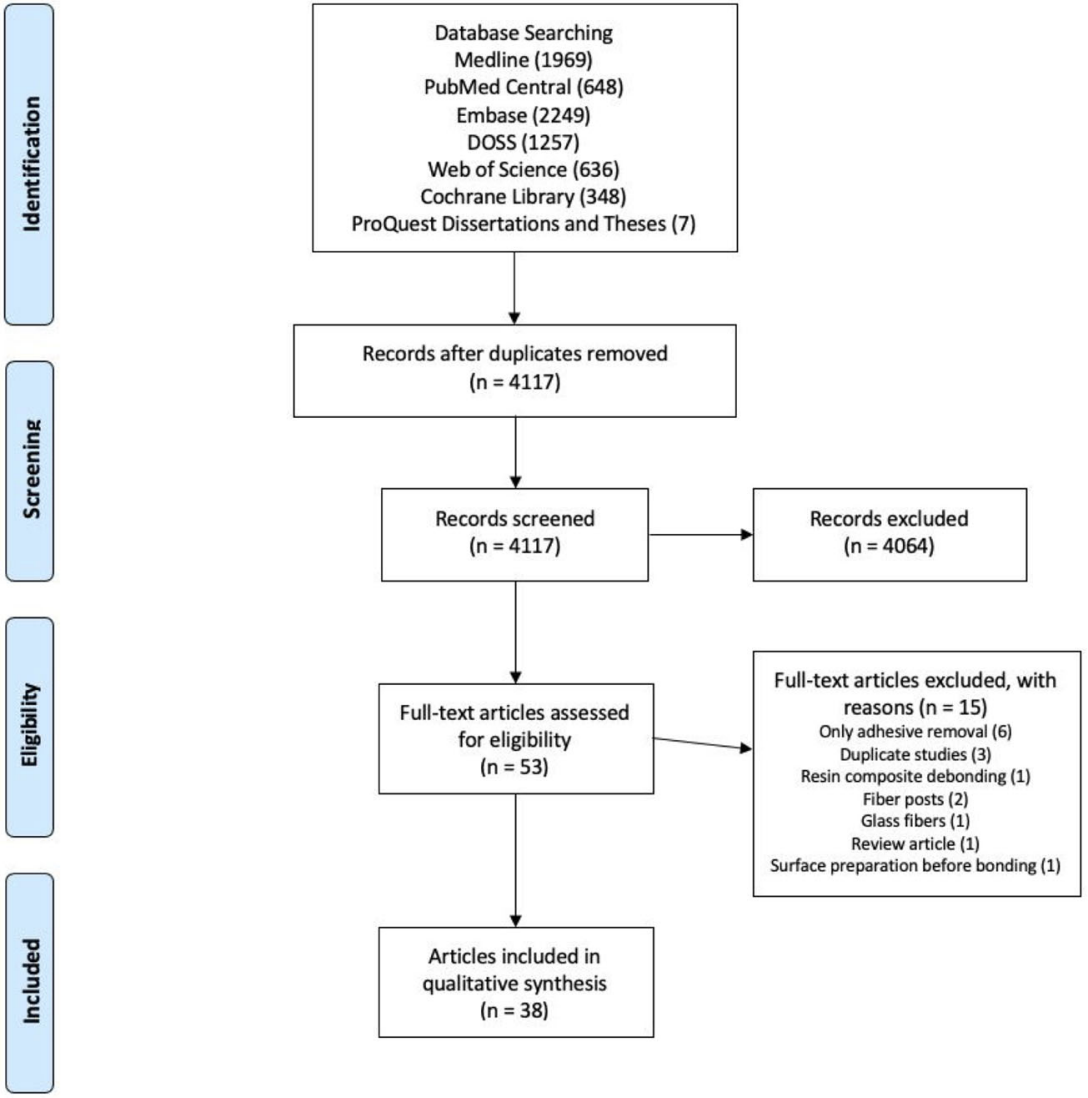
PRISMA flow diagram.

The electronic literature search yielded a total of 7114 articles, of which 4117 were unique citations. The eligible manuscripts were published from 2011 to 2021. Review of titles and abstracts resulted in the exclusion of 4064 records. Fifty‐three full‐text articles were reviewed for inclusion, and 38 were retained for this review. Figure 1 displays the database searches, number of articles retained during each phase of review, and reasons for exclusion. Table 1 provides the detailed search strategies used to search each database.

### Type of samples

Among the 38 manuscripts, 15 reported on experiments conducted with orthodontic brackets (Table 2),^9^, ^10^, ^21^, ^25^, ^26^, ^27^, ^28^, ^29^, ^30^, ^31^, ^32^, ^33^, ^34^, ^35^, ^36^ nine reported on veneer removal (Table 3),^13^, ^37^, ^38^, ^39^, ^40^, ^41^, ^42^, ^43^, ^44^ five studies reported research findings using ceramic disks (Table 4), 6 reported on the all‐ceramic crown removal from natural teeth,^45^, ^46^, ^47^, ^48^, ^49^, ^50^ and 3 studied the removal of the crowns from various implant abutments (Table 5).^7^, ^8^, ^20^ All studies used erbium lasers for ceramic restoration or appliance removal. Six studies conducted experiments with the Er,Cr:YSGG laser, 27 with the Er:YAG of various manufacturers and 5 studies compared the two types of erbium lasers.

**TABLE 2:** The characteristics of included studies on debonding orthodontic brackets

Study	Type of erbium laser	Sample size*	Having no laser control group	Outcome measures	Cement	Type of sample	Examination of restorations	Laser parameters and settings	Summary of study results
Alakus‐SabuncuoGluo 2016^26^	Er:YAG	N=20; extracted human lower incisor (n=10 laser group, n=10 control); ceramic brackets	n=10; no irradiation	Shear Bond Strength (SBS), Adhesive Remnant Index (ARI)	Resin cement (Transbond XT light cure adhesive, 3 M Unitek, Monrovia, CA, USA)	Ortho ceramic brackets (polycrystalline)	N/A	120mJ; 10 Hz; pulse duration:100us; 3 W; contact mode; air/water; time:6s; scanning method “S shape”	3 W power Er: with scanning method demonstrated lower SBS and higher ARI scores during debonding for polycrystalline ceramic brackets
Dostalova 2016^21^	Er:YAG	N=39 (molars); test groups of n=30 divided into 3 subgroups; n=10 ceramic brackets with 3 M Unitek; n=10 metal brackets with 3 M Unitek; and n=10 ceramic brackets with Variolink II	n=9, no laser irradiation, only orthodontic plier	Temperature (thermal infrared camera)	Resin cement (Transbond XT light cure adhesive, 3 M Unitek, Monrovia, CA, USA)	Ortho brackets	SEM	280mJ; 6 Hz; pulse duration:250us; 1.7 W; tip diameter:1 mm; air/fluid10mL/min; time:140s	It is easier to remove the bracket after Er:YAG; Temperature rise was limited; SEM showed no damages.
Dostalova 2016^26^	Er:YAG	N=33 (30 premolars and 3 molars) Group1: n=10 ceramic brackets+Unitek; Group2: n=10 ceramic brackets+Variolink; Group3: n=10 metal brackets+Unitek	n=3; no irradiation	Temperature (thermal infrared camera)	Resin cement (Transbond XT light cure adhesive, 3 M Unitek, Monrovia, CA, USA) and Resin cement (Variolink N, Ivoclar, Vivadent; Schaan, Liechtenstein	Ortho brackets	Stereomicroscopy + SEM	280mJ; 6 Hz; pulse duration:250us; 1.7 W; tip diameter:1 mm; air/water 2 mL/min; time:140s	It is easier to remove the bracket after Er:YAG; Temperature rise was limited; SEM showed no damages.
Downarowicz 2020^27^	Er:YAG and Er,Cr:YSGG	N=13 ortho bracket (n=2 metal and n=11 ceramic)	No	Temperature (thermocouple thermographic camera) in 3 steps (prior to laser debonding, during laser debonding, after laser debonding	Resin cement (Transbond XT light cure adhesive, 3 M Unitek, Monrovia, CA, USA)	Ortho brackets (n=11ceramic +n=2 metal) (extracted premolars)	N/A	Er,Cr:YSGG: 185‐190mJ, 25 Hz, 2.78‐2.85 W, 300us pulse duration, tip diameter:0.6 mm water/air 3.5 mL/s, time=5‐25s; Er:YAG: 4 W; 200mJ; 20 Hz; pulse duration:300us; tip diameter:0.8 mm; air/fluid:3.5 mL/s; time:5‐15s;distance 1‐2mm	For bracket removal temperature inside the tooth didn't increase, and even reduced during cooling water while Er:YAG laser irradiation was applied.
Grzech‐Leśniak 2018^10^	Er:YAG	N=55 (n=20 metal and n=35 ceramic brackets); 3 test groups (each n=15); and 1 control group (n=10)	n=10 no laser+plier (n=5 ceramic and n=5 metal brackets)	Pulp temperature and time + ARI	Resin cement (Transbond XT light cure adhesive, 3 M Unitek, Monrovia, CA, USA)	Metal and ceramic bracket (freshly extracted premolars)	SEM + EDS	3.4 W, 170mJ, 20 Hz, pulse duration 300us, tip diameter:0.8 mm; water/air 3 mL/s, irradiation time 6s, non‐contact with distance 1‐2 mm; circular motion for metal brackets and “S”shape and circular motion for ceramic brackets	Scanning method had significantly lower temperature increase (mean 0.83˚C) compared with circular motion around ceramic brackets.
Hamadah 2016^28^	Er:YAG	N=45 premolars (3 groups for n=15) with different pulse duration (50us, 100us and 300us)	No	Temperature of enamel surface (thermal camera) and ARI adhesive remnant index	Resin cement (Transbond XT light cure adhesive, 3 M Unitek, Monrovia, CA, USA	Ceramic bracket (freshly extracted premolars)	N/A	Non‐contact R02C handpiece, 0.9 mm spot size with fixed distance 0.7 cm; water 2 mL/s+Air 2 mL/s, time=6s; 140mJ/30 Hz (average power 4.2 W) with different pulse duration: 50us, 100us and 300us	No statistically significant difference between the pulse duration of 50,100 and 300us However, 100us and 300us are preferred and suggested.
Hoteit 2020^29^	Er:YAG and Er,Cr:YSGG	N=180 (bovine incisors); 15 groups: n=12 each (6 groups for Er:YAG and 8 groups for Er,Cr:YSGG with different laser debonding setting and 2 groups for control with conventional methods without laser irradiation)	Control with no laser irradiation; conventional pliers	SBS	Resin cement (Transbond XT light cure adhesive, 3 M Unitek, Monrovia, CA, USA	Ceramic bracket (freshly extracted upper premolars)	Stereo microscope + SEM (evaluation of enamel topography)	Perpendicular, non‐contact scanning method; Er,Cr:YSGG: 6 groups, n=12 each; MX7 sapphire tip; 0.7 spot size; turbo handpiece; H mode (60us pulse duration) 70% water+30%air, time=6s. Settings: 3 W/20 Hz (38.96J/cm2); 4 W/20 Hz (51.95J/cm2); 4 W/40 Hz (25.97J/cm2); 5 W/20 Hz (64.93 J/cm2); 5 W/40 Hz (32.47j/cm2). Er:YAG: 6 groups n=12; H02 handpiece, 0.9 mm spot size; SSP mode (50us pulse duration)water 32 mL/min+air 6 mL/min;time=6s. Settings: 80mJ/20Hz(12.58J/cm2); 80mJ/40Hz(12.58J/cm2); 100mJ/20Hz(15.72J/cm2); 100mJ/40Hz(15.72J/cm2); 120mJ/20 Hz (18.83J/cm2); 120mJ/40 Hz (18.83J/cm2); 140mJ/20 Hz (22.01J/cm2); 140mJ/40 Hz (22.01J/cm2)	The most optimal settings for Er: group were 120mJ/40 Hz and 80mJ/40 Hz and for the Er,Cr group were 4 W/20 Hz and 5 W/20 Hz.
Ibrahim 2019^30^	Er:YAG	N=60 (human maxillary first premolar) control, n=30; test group n=30	Control with no laser irradiation; conventional occlusal pad debonding pliers	SBS, ARI		Ceramic bracket (freshly extracted upper premolars)	Stereomicro‐scope	5 W; 250mJ; 20 Hz, pulse duration: 100us; time=10s; swiping motion/scanning+50% water cooling; non contact 2 mm distance	The control group had statistically significant higher SBS. ARI was higher in the laser group but not statistically significant.
Mirhashemi 2019^31^	Er:YAG and Er;Cr:YSGG	N=36 (composite blocks Filtek Z250) control n=12; test group n=24, 3 subgroups, n=12, brackets	Control group n=20 without laser	SBS, ARI	Resin cement (Transbond XT light cure adhesive, 3 M Unitek, Monrovia, CA, USA	Maxillary right central incisor ceramic orthodontic brackets (GAC International. Inc., Islandia, NY, USA)	N/A	Er:YAG: 20 Hz, 3 W, energy density 22/28 J/cm2, pulse duration of 100 μs, exposure time 10s, scanning mode, 2‐mm distance, 1 mm tip diameter. Er:Cr;YSGG: tip diameter of 800 μm, 3 W, 22/28 J/cm2 energy density, pulse duration of 60 μs, irradiated manually for 10s, scanning mode, 2‐mm distance	The mean SBS was 17.01 MPa with Er:YAG laser, 18.03 MPa with Er,Cr:YSGG laser, and 16.61 MPa in the control group; the difference of the three groups was not significant. The difference in the ARI scores and enamel and composite cracks was not significant.
Mundethu 2014^32^	Er:YAG	N=20 (human molars and brackets)	No	SEM, ARI	Resin cement (Transbond XT light cure adhesive, 3 M Unitek, Monrovia, CA, USA	Polycrystalline bracket system (Damon Clear; Ormco Corp, Orange, CA, USA)	SEM	600 mJ, 2 Hz, 800 μs pulse duration, full width at half maximum (FWHM), 1.3 mm fiber tip diameter, and no air or water spray. Contact of fiber tip onto the bracket surface.	Using the described laser parameters, 19 of the 20 brackets could be debonded with just one laser pulse, while one bracket required eight pulses. The ARI score was 3 for all specimens. SEM analysis showed no signs of damage to the enamel
Nalbantgil 2011^34^	Er:YAG	N=80 (bovine mandibular incisors) randomly assigned into four groups of n=10, control, 3, 6, and 9 s of lasing durations. N=30 (human premolars) randomly divided into three groups of n=10, as 3, 6, and 9 s of lasing durations.	Control group n=10 without laser	temperature, SBS, ARI	Resin cement (Transbond XT light cure adhesive, 3 M Unitek, Monrovia, CA, USA	Polycrystalline alumina brackets (Transcend series 6000; 3 M Unitek, Monrovia, CA)	N/A	Power of 4.2 W, 140 mJ at 30 Hz and the application tip (1 mm in diameter), positioned perpendicularly, distance 2 mm from the bracket. The laser energy was applied to the study groups by scanning through the surface of the brackets.	The SBS value for the control group was 22.76 MPa, while values for the 3‐, 6‐, and 9‐s study groups were 12.38, 10.75, and 8.81 MPa, respectively. (statistically significant difference was found between the 3 s and 9 s groups). When ARI scores of the groups were compared, statistically significant differences were observed between the 9‐s study group and control and 6s study groups. The temperature increased from 25°C to 26.27 ±0.3°C, 27.79±0.71°C, and 29.59±0.48°C in the 3‐, 6‐, and 9‐s groups, respectively. The 3‐s group revealed a statistically significantly lower increase in temperature than the 6‐s and 9‐s groups.
Nalbantgil 2014^33^	Er:YAG	N=60 (bovine incisors) 3 groups (n=20): no lasing (control), with cooling, and without cooling	Control group n=20 without laser	Temperature, SBS	Resin cement (Transbond XT light cure adhesive, 3 M Unitek, Monrovia, CA, USA)	Polycrystalline alumina brackets (Transcend series 6000; 3 M Unitek, Monrovia, CA)	N/A	Power of 5 W, laser energy was applied on the surface of the brackets for 9 seconds by scanning the surface of the bracket, tip 1 mm diameter, positioned perpendicularly, distance 2 mm from each bracket; water‐cooling (water group) or without water‐cooling (waterless group).	Mean temperature increases of 2.41°C and 4.59°C were recorded for the water and waterless laser groups, respectively. The shear bond strength value for the control group was 22.76 MPa and 10.46 and 6.36 MPa for the water and waterless laser groups, respectively.
Nalbantgil 2018^35^	Er:YAG	N=80 (bovine incisors) control n=20; test group n=60 (3 subgroups of n=20), 4 groups (3, 6, 9 sec laser application)	Control group n=20 without laser	Temperature, SBS, ARI	Resin cement (Transbond XT light cure adhesive, 3 M Unitek, Monrovia, CA, USA)	Polycrystalline alumina brackets (Transcend series 6000; 3 M Unitek, Monrovia, CA)	N/A	Pulse repetition rate of 20 Hz, pulse duration of 300 msec, water spray of 40–50 mL/min, tip diameter of 1 mm, and the laser irradiation for the three study groups were set at a power of 2 W(100 mJ at 20 Hz), 4 W (200 mJ at 20 Hz), and 6 W (300 mJ at 20 Hz).	Statistically significant difference in intrapulpal temperature increases, SBS and ARI was observed between the groups. Average increased temperatures of 0.67°C, 1.25°C, and 2.36°C were recorded for the 2, 4, and 6 Watt laser groups. The mean shear bond strength was 21.35 MPa for the control group, whereas they were 8.79, 3.28, and 2.46 MPa for the 2, 4, and 6 Watt laser groups, respectively.
Tozlu 2012^9^	Er:YAG	N=100 (100 human premolars) 5 groups, n=20, 4 test groups, debonding 1 s, 18 s, 30 s, 60 s after irradiation	Control with no laser application group, n=20	SBS, ARI	Resin cement (Transbond XT light cure adhesive, 3 M Unitek, Monrovia, CA, USA)	Polycrystalline ceramic brackets (Transcend series 6000, 3 M Unitek, Monrovia, CA)	N/A	Power of 5 W, Laser energy applied to the surface of the brackets for 6s, scanning motion, 1 mm diameter application tip positioned perpendicularly 2 mm from bracket	SBS was significantly ∼ 10 folds lower in the laser group compared to control. ARI of the groups were not statistically different between groups. Debonding ceramic bracket times were 3 folds lower in the laser group than control.
Yilanci 2017^36^	Er:YAG	N=40 (20 premolars and 20 incisors) with monocrystalline brackets	No, laser debonding, with and without thermal‐cycling (same samples repeated two experiments)	Temperature	Resin cement (Transbond XT light cure adhesive, 3 M Unitek, Monrovia, CA, USA	Monocrystalline brackets (Radiance, American Orthodontics, Sheboygan, WI) removal	N/A	H14 contact handpiece, tip (length: 8 mm, diameter: 1.3 mm) 1.2 W, 600 mJ, 2 Hz, long pulse, and no air or water spray The beam spot size was 0.004225 cm2, the power density was 90.4 W/cm2, and the energy density was 45.2 J/cm2.	With thermocycling, the intrapulpal temperature increase of the central incisors was significantly higher than that of the premolars. Laser irradiation duration was related to temperature increase.

* N: sample size in total; n: sample size for each group.

**TABLE 3:** The characteristics of included studies on debonding veneers

Study	Type of erbium laser	Sample size*	Having no laser control group	Outcome measures	Cement	Type of sample	Examination of restorations	Laser parameters and settings	Summary of study's results
AlBakhli 2018^43^	Er:YAG	N=40; extracted human premolars; 40 veneers (5 groups, n=8 each)	No	Temperature, time, failure mode. Type 1: Adhesive failure between the internal surface of the veneer and the luting resin cement, when most of the resin remained on the tooth surface. Type 2: Adhesive failure between the luting resin cement and the tooth surface, when most of the resin remained on the internal surface of the veneer. Type 3: Cohesive failure within the luting resin cement, when a percentage of the remaining resin on both the tooth and the veneer surfaces were somewhat equal.)	Resin cement (Variolink N, Ivoclar, Vivadent; Schaan, Liechtenstein	Veneers: Lithium Disilicate IPS e.max (Ivoclar, Vivadent; Schaan, Liechtenstein)	Stereomicro‐scope	Settings in groups: A) non‐contact at 360mJ/15 Hz, B) contact at 360mJ/15 Hz, C) non‐contact at 400mJ/10 Hz, D) non‐contact at 270mJ/15 Hz, E) non‐contact at 300mJ/10 Hz. Pulse duration of 100 with water/air cooling ratio of 1:1	All veneers were debonded and samples of the NCM (non‐contact debonding) group had considerably lower debonding time (12.6 seconds) than the CM (contact debonding) samples (96.3 seconds), however, higher changes of temperature in NCM (4.28C) than in CM were observed (2.98C). The failure mode of samples was either type 1 or 3.
Alikhasi 2019^44^	Er,Cr:YSGG	N=57 bovine incisors (n=19 feldspathic ceramics, n=19 lithium disilicate with high translucency and n=19 lithium disilicate with medium translucency	No	Temperature and time	Resin cement (Variolink N, Ivoclar, Vivadent; Schaan, Liechtenstein	0.7 mm thickness veneers E.max press (n=19 feldspathic ceramics, Ceramco IC +n=19 Lithium Disilicate reinforced glass ceramic HT (high translucency) and n=19 LD reinforced glass ceramic MO (medium opacity), shade A2	N/A	25 Hz, 2.5 W, 60 us pulse duration, tip MZ6 diameter:0.6 mm, irradiation distance from 2 mm; scanning method	Time: no significant difference among feldspathic ceramics, lithium disilicate reinforced glass ceramic HT (high translucency) and lithium disilicate reinforced glass ceramic MO (medium opacity). Temperature did not exceed the physiological limit.
Giraldo‐Cifuentes 2020^12^	Er,Cr:YSGG	N=68 (bovine incisors with ceramic discs of 1,2,3,4 mm diameter) 4 groups, n= 17 discs per group	No	SBS	Resin cement (Variolink Esthetic® N LC light curing cement, Ivoclar Vivadent, Schaan, Liechtenstein)	IPS e.max‐Press® LT A1 lithium disilicate (Ivoclar Vivadent, Schaan, Liechtenstein)	N/A	4 W of power, 50 Hz power, irradiation time 60s, 4‐mm distance. The energy density per pulse or fluency applied was 5.33 J/cm2	The thicker veneers showed more resistance to the debonding. The debonding strength for group 3 was the highest (5.62 MPa), followed by group 4 (5.20 MPa), then group 2 (0.85 MPa) and finally group 1 (0.0 MPa). The most frequent type of failure was cohesive failure in cement (CC) for all groups, with 73.53%
Iseri 2014^41^	Er:YAG	N=60 (bovine mandibular incisors) c=30; t=30	Control with no laser irradiation	SBS	Resin cement (Variolink II, Ivoclar, Vivadent; Schaan, Liechtenstein)	Porcelain laminate veneers PLV (bovine mandibular incisors) Empress II	N/A	No water; 5 W (50Hzx100mJ) irradiation time 9s; non contact 2 mm distance; tip 1 mm (diameter); scanning method by Oztoprak perpendicularly	The control group had statistically significant higher SBS.
Karagoz‐Yildirak‐Gozneli 2020^40^	Er:YAG	N=120 (human incisors) control n=60 with 4 subgroups; test group n=60, 4 test subgroups, 2 different materials/veneers placed on dentin or enamel, n=15	4 control groups n=15 without laser	SBS	Resin cement (Variolink N; Ivoclar Vivadent, Schaan, Liechtenstein)	60 leucite ceramic discs (IPS Empress; Ivoclar Vivadent, Schaan, Liechtenstein) and 60 lithium disilicate ceramic discs (IPS e.max Press; Ivoclar Vivadent, Schaan, Liechtenstein)	N/A	Irradiation time 9s; 3 W power, 10 Hz, 300 mJ, 100 μs pulse duration	Significant differences were found between the control and laser‐irradiated groups. While the required SBS values for control groups were between 30.04 and 24.66 MPa, the values for laser‐irradiated groups were between 6.60 and 4.09 MPa. There was no significant difference between the control and rebonded groups
Morford 2011^39^	Er:YAG	N=24 (incisors) c 2 group, n=12 each	No	Transmission values of different ceramic systems with different thicknesses, FTIR analysis, debonding time	Resin cement (RelyX veneer, 3 M Unitek, Monrovia, CA, USA)	Veneer samples (IPS Empress Esthetic, e.max Press HT, Ivoclar Vivadent, Schaan, Liechtenstein)	N/A	10 Hz repetition rate, pulse duration 100 useconds at 133 mJ/pulse for debinding). Energy transmission through veneer material samples was determined using the Er:YAG laser at five different set energies delivered by the laser system (133, 217, 316, 400, and 503 mJ per pulse) with a pulse repetition rate of 10 Hz	The veneers transmitted between 11.5% and 43.7% of the incident Er:YAG energy with lithium disilicate transmitting twice the energy as leucite reinforced ceramic at comparable thicknesses. All veneers were completely removed with an average removal time of 113 s. Underlying tooth structure was not damaged. The debonding mainly occurred at the cement/veneer interface. None of the lithium disilicate veneers fractured during debonding, while 36% of the leucite reinforced ceramic fractured.
Walinski 2021^38^	Er,Cr:YSGG	N=22 extracted human incisors with bonded milled laminate veneers	No control group	Time, temperature	Resin cement (Variolink Esthetic LC light‐curing, Ivoclar Vivadent, Inc, Amherst, New York, USA),	Leucite reinforced glass ceramic veneers (Empress CAD, Ivoclar‐ Vivadent, Schaan, Liechtenstein),	N/A	Laser parameters: 333 mJ/pulse, 30 Hz, 80% air, 50% water, 600‐μm diameter fiber tip) with laser fiber (600‐μm diameter cylindrical fiber, 6 mm in length) at a fluence of 885.96 J/cm2. The laser fiber tip held directly in contact perpendicular to the surface, and moved slowly, covering the labial surface of veneer while firing.	When the thickness of a veneer increased, more time was necessary to remove a veneer using Er,Cr:YSGG laser. However, increasing thickness did not necessarily result in an increase in pulpal temperature.
Zanini 2021^38^	Er,Cr:YSGG	N=44 enamel slabs with lithium disilicate laminates randomly assigned into 3 groups based on cement	No	Enamel prism damage	3 different resin cements (Variolink Veneer, Ivoclar Vivadent, Liechtenstein, RelyX U200, 3 M ESPE, USA, and RelyX Veneer, 3 M ESPE, USA)		The morphological, optical, and elemental analysis of enamel was performed before cementation and after laser debonding, using SEM, optical coherence tomography (OCT), and EDS.	Two different protocols: 3.5 W, 48.14 J/cm2, 20 Hz non‐contact; and 3.0 W, 48.14 J/cm2, 20 Hz noncontact	There was presence of residual cement in most experimental groups. Morphological analysis showed alteration of the enamel's prisms only in the groups that used RelyX Veneer and Variolink Veneer cements. There was no evidence of deleterious morphological changes resulting from irradiation. However, an increase in the optical attenuation coefficient by the OCT was observed due to the presence of the remaining cement and suggests an increase in temperature during irradiation. It can be concluded that the Er,Cr:YSGG laser is efficient for veneer removal without causing deleterious effects for the enamel.
Zhang 2018^13^	Er:YAG	N=12 (human premolars)	No	Time, pulse number	Resin cements (Single bond 3 M, Espe, USA and RelyX Veneer 3 M Espe,USA)	Porcelain veneers (powder Duceram Kiss, USA) removal	SEM	Non‐contact sapphire tip with air‐water spray used for veneer debonding at 100 mJ energy and 30 Hz Frequency (Fluence 19.94 J/cm2).	The veneer debonding is possible with an Er:YAG laser. The total number of pulses seems not related to the laser efficiency. SEM observation confirms that residual tooth structure was not noticeably altered.

* N: sample size in total; n: sample size for each group.

**TABLE 4:** The characteristics of included studies on debonding ceramic disc specimens

Study	Type of erbium laser	Sample size*	Having no laser control group	Outcome measures	Cement	Type of sample	Examination of restorations	Laser parameters and settings	Summary of study results
Culhaoglu 2021^53^	Er:YAG	N=120 (human upper central and lateral teeth) Three different materials, 6 groups (n=18, 3 with laser, 3 without laser‐control) Two thickness 0.5 and 1 mm, 4 group (n=27 each for different thickness with laser and without)	n=3; no irradiation	SBS	Resin cement (Rely X Veneer, 3 M ESPE, St Paul, MN, USA)	Feldspar ceramic (Vita Cerec Blocks; Zahnfabrik), lithium disilicate glass ceramic (IPS e.max; Ivoclar‑Vivadent), and resin nanoceramic (Lava Ultimate; 3 M ESPE) specimens	Scanning Electron Microscopy (SEM)	Irradiation distance 2 mm from the specimen surface. 150 mJ pulsation energy, 10 Hz pulsation frequency, 100 micro seconds pulsation width, and 1.5 W power. The laser application equipment used the 60% water and 40% air mode for 9 s	The laser application was found to weaken the SBS values; however, the highest SBS decrease was observed for laser treatments for all different sample thicknesses.
Oztoprak 2012^52^	Er:YAG	N=80 (bovine incisors) control and 3 test group (n=20) of 2, 4, 6 W power	Control group n=20 without laser	SBS	Resin cement (Variolink II, Ivoclar Vivadent, Schaan, Liechtenstein)	Lithium disilicate ceramic discs (IPS Empress II, Ivoclar Vivadent, Schaan, Liechtenstein)	N/A	Power of 5 W (50 Hz×100 mJ), 1 mm tip, 2‐mm distance from the laminate veneers. Scanning was performed with horizontal movements parallel to the surface.	The mean SBS value for the control group was 27.5 ±1.44 MPa, while values for the 3‐, 6‐, and 9‐s study groups were 10.58 ±0.9, 8.47 ±0.8, and 3.54 ±0.46 MPa, respectively.
Rechman 2014^5^	Er:YAG	N=15, 3 groups of n=5, different bonding cements.	No	Transmission Values of Different Ceramic Systems with Different Thicknesses, FTIR analysis	Resin cements (Variolink Veneer, Variolink II, Multilink Automix, or SpeedCEM, Ivoclar Vivadent, Schaan Liechtenstein)	IPS Empress Esthetic (EE) (leucite glass ceramic), IPS E.max CAD LT A2 (LS2) (E.max CAD), and IPS E.max ZirCAD crowns (Ivoclar Vivadent, Schaan, Liechtenstein)	N/A	10 Hz repetition rate, pulse duration 100 us at 126 mJ/pulse to 300 us at 508 mJ/pulse)	All bonding cements showed a broad H2O/OH absorption band. Leucite reinforced ceramics and lithium disilicate CAD ceramics transmitted between 21 and 60% of the incident energy, with E.max CAD transmitting more energy than EE at comparable thicknesses. IE.max ZirCAD transmitted only 5−10% of the incident energy. Initial signs of cement deterioration occurred at 1.3−2.6 J/cm2. Multilink Automix, SpeedCEM, and Variolink II started ablation at 4.4−4.7 J/cm2. Variolink Veneer needed 44% less energy for ablation.
Sari 2014^54^	Er:YAG	N=10	5 test groups, 2 discs per group	Transmission values of different ceramic systems with different thicknesses, with power meter	No cement	10 discs, with 0.5, n=5 and 1 mm, n=5 thickness (zirconium‐oxide core ceramic, monolithic zirconium‐oxide ceramic, feldspathic ceramic, leucite‐reinforced glass ceramic, and lithium disilicate‐reinforced glass ceramic)	N/A	1 W (500 mJ, 2 Hz) power output. A contact handpiece of the laser device (H14‐C, Fotonacd.d.) and a sapphire tip 1.3 mm in diameter and 8 mm.	Transmission ratio through the ceramic material decreased with increasing thickness of the ceramic samples. The highest transmission ratio was determined for lithium disilicate‐reinforced ceramic with 0.5 mm thickness (88%), and the lowest was determined for feldspathic ceramic with 1 mm thickness (44%).
Tak 2015^51^	Er:YAG	N=10	5 test groups	The volume of the resin cement discs was measured using a micro‐CT system (SkyScan, Bruker microCT, Kontich, Belgium) before and after the Er:YAG laser irradiation.	5 different resin cements: G‐Cem LinkAce (GCCorp., Tokyo, Japan), Multilink Automix Variolink II (Ivoclar Vivadent, Schaan, Liechtenstein), Panavia F 2.0 (Kuraray Noritake Dental, Tokyo, Japan), RelyX Unicem U100 (3 M ESPE, St. Paul, MN)	The ceramic discs,5 mm lithium‐disilicate discs (IPS e.max CAD/A2‐HT, Ivoclar‐ Vivadent, Schaan, Liechtenstein)	Micro‐CT	Contact handpiece (R14), distance 1 mm. A sapphire tip of 1.3 mm diameter and 8 mm length used for the irradiation. The output of the laser device was set to 600mJ 2 Hz (1.2 W) and 1,000 ms (VLP Very Long Pulse) pulse duration (Energy density 45.4 J/cm2)	Cement volume loss was determined. The highest volume loss was determined for G‐Cem (1.1 ±0.6mm^3^) and Multilink (1.3 ±0.1mm^3^) groups, and the lowest volume loss was determined in Rely X (0.3 ±0.07mm^3^), Variolink (0.4 ±0.2mm^3^), and Panavia (0.6 0.2mm^3^) groups.

* N: sample size in total; n: sample size for each group.

**TABLE 5:** The characteristics of included studies on debonding ceramic crowns

Study	Type of erbium Laser	Sample size*	Having no laser control group	Outcome measures	Cement	Type of sample	Examination of restorations	Laser parameters and settings	Summary of study results
Deeb 2020^49^	Er,Cr:YSGG	N=24 (n=12 primary and n=12 permanent teeth), 24 all‐ceramic zirconia crowns debonding. Three experiments ‐ different cements and laser settings	No	Temperature and time	Resin modified glass ionomer (RMGI) cement (BioCem; NuSmile, Houston, TX, USA) or Resin Cement (RelyX (RelyX Luting Plus Automix Resin Modified Glass Ionomer Cement; 3 M ESPE: St. Paul, MN, USA)	Prefabricated zirconia crowns (NuSmile, Houston, USA)	N/A	Two laser settings: 4.5 Watts, 15 Hertz, 20 water/20 air, and 5 Watts, 15 Hertz, 50 water/50 air	The average time for crown removal was: 3 min, 47.7 s for permanent; and 2 min 5 s for primary teeth. The mean temperature changes were 2.48 ±1.43°C for permanent; and 3.14 ±1.88°C for primary teeth. The time to debond was significantly positively correlated with tooth inner surface area and volume, outer crown volume, and the cement volume.
Deeb 2021^50^	Er:YAG and Er,Cr:YSGG	N=25 (25 permanent molars), 25 all‐ceramic zirconia crowns, 2 retrieval treatment groups: Er:YAG laser group (G1; n = 12) or the Er,Cr:YSGG laser group (G2; n = 13).	No	Temperature and time	Resin modified glass ionomer (RMGI) cement (BioCem; NuSmile, Houston, TX, USA)	Prefabricated zirconia crowns (NuSmile, Houston, USA)	SEM	Er:YAG laser: 300 mJ, 15 Hz, 4.5 W, and 50‐microsecond pulse duration (SSP mode); Er,Cr:YSGG laser: 4.5 W, 15 Hz, 20 water/20 air, and 5 W, 15 Hz, 50 water/50 air, and 60‐microsecond pulse duration (H mode)	The average time for crown removal using the Er:YAG laser was 1 min 32.7 s; for the Er,Cr:YSGG laser it was 3 min 13.9 s. The mean temperature changes were 1.41 ±1.36°C for the Er:YAG laser and 2.2 ±0.99°C for the Er,Cr:YSGG laser. The SEM examination showed no damage or major structural changes.
Deeb 2019^8^	Er:YAG	N=20 identical lithium disilicate implant crowns cemented onto zirconia prefabricated implants abutments	no	Temperature and time	Resin cement (Variolink N, Ivoclar, Vivadent; Schaan, Liechtenstein	Custom CAD‐CAM lithium disilicate crowns (IPS E.max, Ivoclar Vivadent, Schaan, Liechtenstein) from zirconia prefabricated abutments (Zimmer Biomet Dental, Palm Beach Garden, FL, USA)	SEM + Secondary Electron Imaging (SEI) + Back‐Scattered Electrons (BSE)	H02 non contact handpiece with distance 10 mm, spot 0.9 mm, irradiation time 30‐60 sec per surface, no irradiation for occlusal surface; 300mJ, 15 Hz, 4.5 W, QSP mode; water/air 2/2	Time for ceramic crown removal from zirconia abutments were 4 minutes (min) and 42 second to 3 min 12 sec The temperatures during irradiation ranged from 18.4˚C to 20˚C and 22.2˚C to 24.5˚C for the abutment and the crown during irradiation from 1 min to 10 mins.
Elkharashi 2020^20^	Er:YAG and Er,Cr:YSGG	N=20 zirconia monolithic implant crown (n=10 Er:YAG and n=10 Er,Cr:), zirconia abutments	No	Temperature and time	Resin cement (Rely X Unicem2, 3 M ESPE: St. Paul, MN, USA)	Zirconia monolithic crown 1‐2 mm thickness (IPS E.max ZirCAD, Ivoclar Vivadent, Schaan, Liechtenstein) from zirconia prefabricated implant abutments, (Zimmer Biomet Dental, Palm beach Garden, FL, USA) + recementation and debonding	SEM + Energy‐Dispersive X‐ray Spectroscopy (EDS)	Er:YAG: 300mJ, 15 Hz, 4.5 W, SSP mode (50us pulse duration), water/air 2/2; Er,Cr:YSGG: 4.5 W, 15 Hz, operation mode: H (60 μs pulse duration), 20 water/20 air	The average times of zirconia crown removal from zirconia abutments were 5 min 20 sec and 5 min 15 sec for the Er:YAG laser of first and second experiments, and 5 min 55 sec for the Er,Cr:YSGG laser experiment
Grezch‐Lesniak 2020^48^	Er:YAG	N=26, n=13 zirconia and n=13 lithium disilicate crowns on extracted human molars	No	Temperature and time	Resin cement (Variolink Esthetic DC dual curing, Ivoclar Vivadent, Schaan, Liechtenstein)	CAD‐CAM lithium disilicate crowns (IPS E.max, Ivoclar Vivadent, Schaan, Liechtenstein) and zirconia (IPS E.max ZirCAD, Ivoclar Vivadent, Schaan, Liechtenstein) crowns	SEM	300mJ, 15 Hz, 4.5 W, QSP mode; water/air 2/2; H02 non‐contact handpiece, distance 10 mm, spot size 0.9 mm, irradiation time 30‐60 sec per surface, no irradiation for occlusal surface	Time for zirconia is longer than lithium disilicate. SEM showed no damages. Temperature change was within physiological limit. The retrieval times were 267.1±130.43, 220± 79.09, 277.1±126.44, 368.4± 136.14, 355±159.39, and 419.2 ±121.36 s for first, second, third lithium disilicate and zirconia groups, respectively. The maximal temperatures (˚C) were 23.95.1±1.89, 24.86 ± 2.01, 24.17± 1.53, 22.88 ±1.51, 24.03±1.74, and 21.99± 1.32 for first, second, third lithium disilicate and zirconia groups,
Grzech‐Leśniak 2019^7^	Er:YAG	N=40 lithium disilicate implant crowns, titanium abutments (n=20 resin cement, n=20 resin modified glass ionomer cement)	No	Temperature and time	Resin cement (Variolink Esthetic DC dual‐curing, Ivoclar Vivadent, Schaan, Liechtenstein) and resin‐modified glass ionomer(Fujicem II LC, GC America, Alsip, IL, USA)	CAD‐CAM lithium disilicate crowns (IPS E.max, Ivoclar Vivadent, Schaan, Liechtenstein) from titanium prefabricated implant abutments (Zimmer Biomet Dental, Palmbeach Garden, FL, USA)	SEM + BSE	Noncontact HO2 handpiece, distance 5‐10 mm, water/air 2/2; 300mJ; 15 Hz; 4.5 W; mode:QSP; spot 0.9 mm, continuous motion; no irradiation on occlusal surface; irradiation time 30s+30s+	Average times were 196.5 sec for CR and 97.5 sec for RMGI; temperature change was within physiological limit, SEM showed no damage.
Gurney 2016^47^	Er,Cr:YSGG	FIRST study; N=45; checking optimal parameters with range of Wattage: n=20 molars, 20 lithium disilicate discs (n=5 control+ n=20 Er,Cr divided into 4 groups); SECOND study: N=25 anterior and premolar, 25 lithium disilicate crowns with 3.5 and 4 W; n=20 laser debonding, n=5 drill(control)	Control n=5 (air rotary high speed (n=3), electric (n=2) and bur+pliers LASER: n=20 + n=25	Time	Resin resin (Multilink Automix, Ivoclar Vivadent, Schaan, Liechtenstein)	CAD‐CAM lithium disilicate crowns (extracted molars) 1.5 mm of thickness + 25um space	N/A	FIRST study: 4 different wattages: 3 W; 3.5 W; 4 and 5 W; water 60%+30%air; 25 Hz; time=30s+30s+; SECOND study: n=25 premolars+anterior; Group 3.5 W with time=60s and 90s and Group 4 W, time=30s and 60s; 25 Hz; continuous motion without proximal surfaces	The goal of the study was to establish good parameters for debonding. Results: 3.4 and 4 W was the best; specimens scanned with 3 W couldn't be removed
Rechman 2014^6^	Er:YAG	N=40 (human molars), 20 lithium disilicate and 20 zirconia crowns	No	Time	Resin cement (Ivoclar Multilink Automix,Ivoclar Vivadent, Schaan Liechtenstein)	IPS E. max CAD shade LT A2 (LS2) (E.max CAD) and IPS E.max ZirCAD shade MO0 (ZrO2) (ZirCAD) (Ivoclar, Vivadent, Liechtenstein).	N/A	1.100 mm diameter fiber tip with energies up to 600mJ per pulse, 10 Hz, pulse duration 100us at 126 mJ/pulse, and 400us at 590mJ/pulse). Distance of 10 mm from the crown surface. Air‐water spray 67 ml/minute.	All of the all‐ceramic crowns were successfully debonded with the laser. On average, an all‐ceramic lithium disilicate CAD crown was debonded in 190 s. The debonding time for zirconia feather‐edge crowns was 226 s and for zirconia crowns with regular margin was 312 s. No crowns fractured and no damage to the underlying dentin was detected. The bonding cement deteriorated due to the Er:YAG irradiation.
Rechman 2015^46^	Er:YAG	N=20 (human molars) with lithium disilicate crowns	No	Temperature, time	Resin cement (Ivoclar Multilink Automix,Ivoclar Vivadent, Schaan Liechtenstein)	E.max CAD full contour crowns (Ivoclar Vivadent, Schaan, Liechtenstein)	N/A	10 Hz repetition rate, and a pulse duration of 400 μs at a 590 mJ pulse, tip with 1100 μm diameter. Fluence of 45 J/cm2 at the ceramic surface (5° average beam divergence); distance of 5 mm from the crown with air–water spray.	Average debonding time of 135 ±35 s. No crown fractured, and no damage to the underlying dentin The temperature rose in the pulp chamber on averaged of 5.4 ±2.2°C.

* N: sample size in total; n: sample size for each group.

Comparisons included: ceramic crowns on natural teeth, ceramic crowns on various implant abutments and veneers. All studies were assessed for the type of erbium family laser. In all studies, including repeated experiments, a total of 80 ceramic crowns were debonded from implant abutments and 160 ceramic crowns were debonded with erbium lasers from teeth. Of the veneer samples, 355 debonded, while 183 ceramic disc samples debonded.

### Identified outcome measures in the included studies

The outcome measures of the included studies are demonstrated in Tables 2, 3, and 5. Debonding time and pulpal or abutment temperature changes were perhaps the most common outcome measures used in determining the effectiveness of laser assisted debonding. A majority of the studies measured both debonding time and temperature changes.^7^, ^8^, ^10^, ^20^, ^38^, ^44^, ^46^, ^48^, ^49^, ^50^ Laser transmission energy was also used in relation to the laser application.^39^, ^54^ Shear bond strength (SBS), adhesive remnant index (ARI), and mode of failure were also examined in several studies.^9^, ^10^, ^12^, ^26^, ^28^, ^29^, ^30^, ^31^, ^32^, ^33^, ^34^, ^35^, ^40^, ^41^, ^52^, ^53^ One study examined the laser pulse values^13^ and another examined the remaining cement volume.^51^ One study examined the enamel prism damage.^38^ However, several studies examined the abutment specimens and ceramic specimens using scanning electron microscopy (SEM) and/or energy dispersive X‐Ray spectroscopy (EDS) and/or optical microscopy.^7^, ^8^, ^10^, ^21^, ^26^, ^29^, ^30^, ^32^, ^48^, ^50^, ^53^ Secondary analyses such as scanning electron imaging (SEI) + Back‐scattered electrons (BSE) might also be used.^7^, ^8^ One study used micro‐computed tomography (micro‐CT).^51^

### Relationship between restorative or luting materials and debonding time

Based on reported data, type of cement plays a role in the debonding time and has been associated with structural changes seen on microscopic and electron microscopic analyses.^7^, ^8^, ^10^, ^13^, ^20^, ^21^, ^26^, ^29^, ^30^, ^32^, ^38^, ^43^, ^48^, ^50^, ^53^ While Er,Cr:YSGG laser was deemed efficient for veneer (Table 3) removal without causing deleterious effects to the enamel, presence of residual cement and changes in enamel prism structure were observed in association with certain resin cements (RelyX Veneer, 3M‐ESPE and Variolink Veneer, Ivoclar Vivadent).^37^ Resin cements in general are greatly affected by the erbium laser irradiation, however the ablation thresholds and ablation volume loss are dependent on the chemical composition of a particular resin cement. Some cements, such as was determined in G‐Cem LinkAce, GC America, and Multilink N, Ivoclar Vivadent cements, exhibit higher volume loss compared to other such as Rely X Unicem U100, 3M‐ESPE, Variolink II Ivoclar, and Panavia F‐20, Kuraray Dental, which appear to have less volume loss.^51^ Debonding of lithium disilicate crowns from titanium implant abutments requires a shorter time when crowns are cemented with a resin‐modified glass‐ionomer cement (97.5 seconds) versus resin cement (196.5 seconds).^8^ Thicker veneer restorations show more resistance to debonding.^42^ Translucency of the same type of ceramic appears to have little influence on debonding. No significant difference in debonding veneers from bovine incisors was reported among feldspathic ceramics with different translucency.^44^ While the translucency of the ceramic plays a nonsignificant role in the laser transmission and debonding, the thickness of ceramic appears to be critical to the laser transmission and thus the debonding efficiency. Transmission ratio through the ceramic material decreased with increasing thickness of the ceramic sample. The highest transmission ratio was determined for lithium disilicate‐reinforced ceramic with 0.5 mm thickness (88%), and the lowest was determined for feldspathic ceramic with 1 mm thickness (44%).^54^ The details on debonding time are discussed below.

Time to debond a crown has been positively correlated with surface area and volume of the tooth and crown, and the volume of the luting cement.^49^ The larger abutment surface area was positively correlated to the longer retrieval time.^48^ Laminate veneers with an average thickness at the midfacial of 0.75 mm can be removed in 14.16 ±0.60 seconds using Er,Cr:YSGG laser.^38^ As the thickness of a veneer increases, more time is necessary to remove it.^38^ Time for debonding varies for all‐ceramic crowns depending on the material. Studies reported that it takes longer to debond a zirconia crown (226‐312 seconds) compared to a lithium disilicate crown (190 seconds) from a human molar.^6^, ^48^ This has also been reported for debonding of all‐ceramic crowns from implant abutments. Removal of zirconia crowns from zirconia abutments took on average up to 355 seconds,^20^ while debonding of lithium disilicate crowns can be accomplished in shorter time from zirconia abutments (192‐181 seconds),^8^ and titanium abutments (97.5‐196.5 seconds).^7^

### Pulpal temperature changes

Several manuscripts measured pulpal temperature changes during laser irradiation.^27^, ^36^, ^38^, ^49^ No study reported a significant increase in pulpal temperature beyond the physiological limit of 5.5°C during irradiation with any erbium laser. Irrigation used during the debonding process may sometimes even lower the pulpal temperature.^27^ Pulpal temperature changes ranged between 0.71°C to 4.28°C. For bracket removal, the temperatures increased 0.83°C, while for crown removal temperature increased up to 4.5°C. The temperature increases may also be influenced by the proximity of the pulp chamber to the irradiated surface and the thickness of the remaining tooth structure. The intrapulpal temperature increase in central incisors was significantly higher than that of the premolar teeth when removing brackets with Er:YAG laser at 1.2 W, 600 mJ, 2 Hz.^36^ A similar trend was observed when debonding prefabricated zirconia crowns from primary teeth with larger pulps and thinner layer of dentin and thus closer proximity to the irritated surface.^49^ Increasing thickness of veneer restoration does not necessarily result in an increase in pulpal temperature despite requiring longer time of irradiation for debonding.^38^

### Type of the erbium laser and settings

Five studies compared the efficiency of debonding between the Er,Cr:YSGG and Er:YAG lasers. Time for removal was not always statistically different. However, the debonding times in general tend to be shorter for the Er:YAG laser compared to the Er,Cr:YSGG laser.^20^, ^27^, ^29^, ^31^, ^50^ Both erbium lasers feature comparable times to debond orthodontic brackets (Table 2),^10^, ^29^, ^31^ and do not result in the temperature increase inside the tooth.^27^ While both erbium lasers are efficient in removing zirconia crowns from teeth or implant abutments, Er:YAG laser accomplishes the task faster than Er,Cr:YSGG.^20^, ^50^ While power of 3 Watt, 10 seconds in the scanning mode with energy density 22‐28 J/cm2, pulse duration of 100 μs seems to be adequate for orthodontic bracket removal,^31^ crown removal requires laser settings to be in the range of at least 3.5 and 4 Watt of power with 25 Hz pulse rate.^47^ Most studies used settings between 4 and 6 Watt. Power of 5 Watt; 100‐250 mJ, 20‐50 Hz has been used for veneer debonding^41^ and orthodontic bracket removal,^30^ while 4.5 Watt, 300 mJ, 15 Hz was reported to be efficient and safe for removal of lithium disilicate and zirconia crowns from implant abutments^7^, ^8^, ^20^ and teeth.^48^

### Examinations of irradiated ceramics and sample surface analyses

Most studies reported successful removal of restorations or appliances with no detectable physical damage. All of the all‐ceramic crowns were successfully debonded from teeth or implant abutments with the erbium lasers. No crowns were fractured and no damage to the underlying abutment was detected. The bonding/luting cement was usually deteriorated due to the erbium laser irradiation. Veneer debonding with the Er:YAG laser at 100 mJ and 30 Hz resulted in intact underlying tooth structure.^13^

Fifteen out of 38 studies (Tables 2, 3, 4, and 5) examined the debonded ceramics. Scanning electron microscopy (SEM) was the most common method of examination.^7^, ^8^, ^10^, ^13^, ^20^, ^21^, ^26^, ^29^, ^37^, ^48^, ^50^, ^53^ SEM was sometimes applied with energy dispersive spectroscopy (EDS),^20^, ^37^ secondary electron imaging (SBI), or backscattered electrons (BSE).^10^ EDS provides chemical compositions of materials, while SBI and BSE provide further details on ceramic cracks and surface structural damages. It is particularly useful to see that the composition of the debonded ceramic materials, either ceramic crowns or implant abutments, appeared to have no chemical or structural damages from erbium laser application for debonding. Multiple repeated laser debonding appeared to have little to no effect on the ceramic surface structure.^20^ Results from all studies showed that erbium lasers are effective in debonding all ceramic restorations with no damage to abutment teeth/implant and with none or minimal alterations to the ceramic restorative/orthodontic appliance surfaces.

## Discussion

**FIGURE 2:**
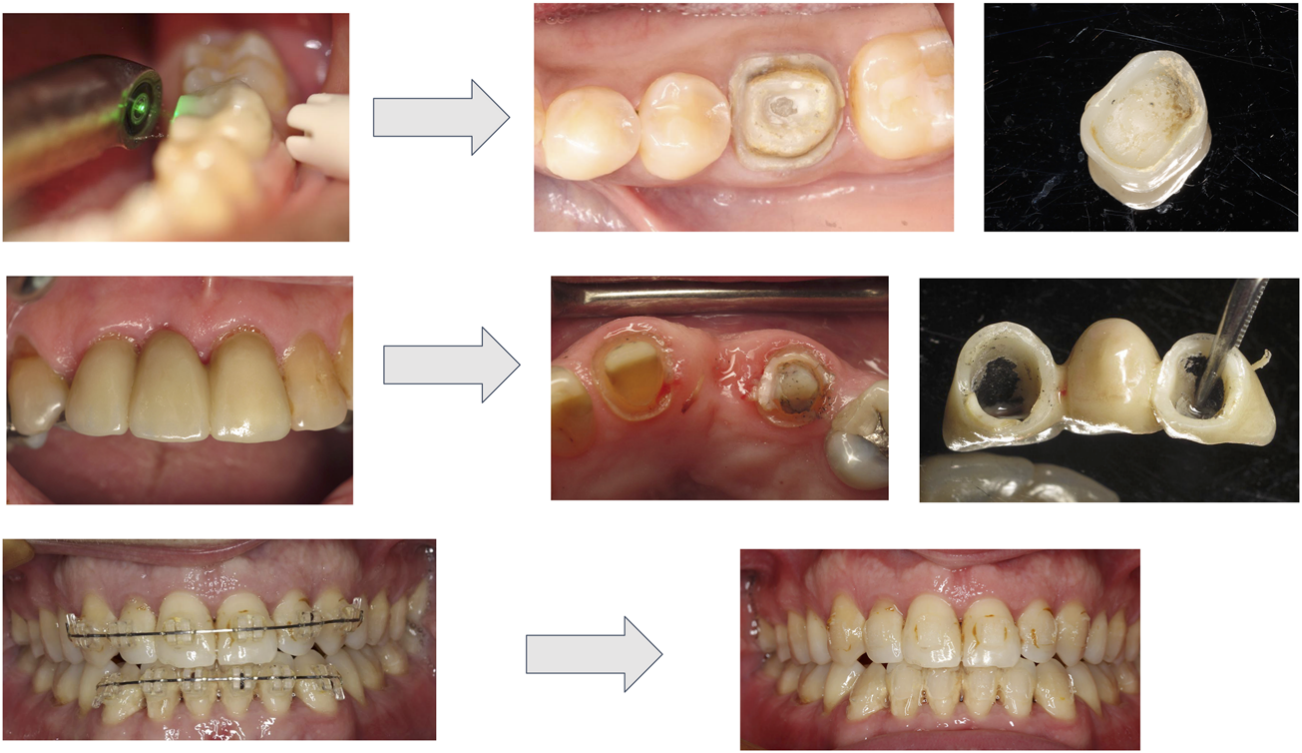
Clinical examples of laser debonding using Er:YAG laser; (top) Debonding of an all‐ceramic lithium disilicate crown; (middle) Debonding of a monolithic zirconia fixed partial denture; (bottom) Debonding of orthodontic brackets.

This study is one of the first to comprehensively review contemporary research on ceramic debonding using erbium lasers. Results from this scoping review revealed that erbium lasers have been reported as an option to remove ceramic restorations from natural teeth and dental implant abutments. An orthodontic bracket appliance or a ceramic crown can be removed without any damage to the natural tooth or implant abutments in a few minutes. The light energy emitted by erbium lasers transmits through the translucent ceramic materials and is selectively absorbed by water molecules and residual monomers in cements resulting in cement ablation and hydrodynamic ejection.^5^, ^7^ The bond between the ceramic restoration and the tooth or implant abutment is disrupted mainly within the cement layer and at the ceramic/cement interface (Fig 2).

A majority of the included studies were performed on extracted natural teeth, thus data obtained in clinical in vivo trials is lacking. In this scoping review, there was no in vivo or in human studies included because only case reports or case series were currently present in the literature search. The most robust evidence is available for debonding of orthodontic brackets which is based on human and bovine extracted teeth. The erbium laser irradiation capacity to remove and degrade composite resin materials is applied for the removal of bonded ceramic materials to tooth structure.^6^ The improvement of ceramic‐natural tooth bonding technology presents a challenge to clinicians. In addition, removal of a well‐fitted implant supported cement‐retained fixed prostheses from customized implant abutments can also be a clinically challenging task. Only a few ex vivo studies have examined the application of erbium lasers for this implant prosthesis removal purpose.^7^, ^8^, ^20^ The optimal laser power settings for a majority of ceramic restorations/appliances have been reported in the range of 4.0 to 5.5 Watt.^8^, ^12^, ^20^, ^41^, ^47^, ^48^

It appears that the time required to debond ceramic restorations correlates to the volume of the luting cement,^49^ the size of the abutment surface area,^48^ thickness and characteristics of the restorative materials,^6^, ^38^, ^42^, ^48^ and chemical composition of the luting cement.^37^ In general, it takes longer to debond zirconia than lithium disilicate crowns from either natural teeth or implant abutments.^7^, ^8^, ^20^ The zirconia material which is mainly crystalline structure is possibly less penetrable compared to lithium disilicate material which has more matrix in the structure.^20^ Resin and glass‐ionomer cements display different ablation thresholds and ablation volume loss based on their chemical composition,^51^ resulting in different debonding efficiency of the restoration.^8^ This is likely due to various amounts of water molecules and unpolymerized monomers left in the cements. Translucencies of ceramics seem to have no significant effect on time required to debond veneers.^44^

The safety concern of raising pulpal temperature while using the erbium lasers to facilitate restorative debonding has been studied. The increase in pulpal temperature did not exceed 5.5°C, which could cause thermal injury to the pulpal tissue.^22^ The temperature raised in all studies pointed to the safety of erbium lasers to the pulpal tissue,^22^ periodontium,^24^ and bone.^23^ None of the studies reported significant problems with intrapulpal temperature increases during laser irradiation using the proposed settings. Furthermore, many studies examined the debonded ceramics using microscopy, scanning electron microscopy (SEM), and energy dispersive x‐ray analysis (EDX) and reported no detectable damage to the abutment and ceramic surface.^7^, ^8^, ^20^ It can thus be suggested that laser‐assisted ceramic restoration removal does not present a thermal risk for the tooth or implant nor does it damage the ceramic restorative material.

While both erbium lasers are efficient in debonding restorations, the Er,Cr:YSGG laser may take longer due to its lower absorption coefficients compared to the Er:YAG laser.^20^, ^27^, ^29^, ^31^, ^50^ The Er,Cr:YSGG laser wavelength penetrates deeper into the tissue and requires more time to heat up to the evaporation temperature, while the substance heated by the Er:YAG laser will reach ablation temperatures faster.^55^ More importantly the absorption peak to the water molecule of the Er:YAG laser is slightly closer than the peak of Er,Cr:YSGG laser, therefore the Er:YAG laser appears to be more proficient than Er,Cr:YSGG laser in ceramic debonding.^20^ However, the two erbium pulsed lasers may be used interchangeably for debonding of ceramic restorations and brackets despite having different wavelengths, as both of them target water as their chromophore.

It is important to note the limitations of this scoping review. First, all studies included in this review were ex vivo or simulated. Extracted human and bovine teeth, as well as implant fixtures, were used in typodont simulated or other models. This may not be reflective of clinical situations where the patient's oral structures, tongue and cheek, as well as optimal access to the laser intraorally, can present challenges in manipulating the laser handpiece and application. Debonding time in clinical application may likely take longer than the simulated debonding time. More importantly, there is a real need for clinical studies to demonstrate the in vivo‐based debonding time as well as to assess patient's compliance and acceptance of the laser assisted ceramic debonding. In addition to the fact that all studies in this scoping review are in vitro, the in vitro procedures were varied and not standardized. For example, some studies utilized manikins to simulate a clinical setting, others did not or did not report the use of a typodont or manikin. It is likely that in the clinical setting the time of retrieval would be longer. Therefore, a meta‐analysis was not performed which would have been appropriate if more clinical studies were available. Future prospective controlled clinical trials are recommended. Second, there is no standardized protocol for laser settings in restorative/orthodontic appliance removal. While this scoping review has shown the consistency in the similar applications of laser settings, there is a need for a consensus for this procedure for both Er:YAG and Er,Cr:YSGG lasers. Finally, this scoping review only included English‐language peer‐reviewed literature. Attempts were made to review theses/dissertations, conference papers, as well as other sources of gray literature. However, no study was found relevant to this scoping review. It is possible that there are some unpublished works that may have been missed.

## Conclusion

Erbium lasers present a noninvasive protocol for retrieval of ceramic restorations/appliances from teeth and implant abutments. Laser‐assisted ceramic restoration and orthodontic bracket removal provides efficient restoration retrievability without damage to the material or the abutment surface. While the majority of the current literature did not compare the laser method to conventional rotary instruments, the laser method is a safe, predictable, and time efficient alternative to rotary‐assisted crown removal. Clinicians need to possess adequate knowledge regarding laser safety and parameters with reference to different tissues and ceramics. Further well‐designed controlled clinical trials and longitudinal prospective studies are needed to determine the safest and most effective laser parameters for irradiation of ceramic restorations with varying thicknesses and luted with cements of various chemical compositions.
